# Development of nomograms to predict axillary lymph node status in breast cancer patients

**DOI:** 10.1186/s12885-017-3535-7

**Published:** 2017-08-23

**Authors:** Kai Chen, Jieqiong Liu, Shunrong Li, Lisa Jacobs

**Affiliations:** 1 Guangdong Provincial Key Laboratory of Malignant Tumor Epigenetics and Gene Regulation, Sun Yat-Sen Memorial Hospital, Sun Yat-Sen University, Guangzhou, China; 2 Breast Tumor Center, Sun Yat-sen Memorial Hospital, Sun Yat-sen University, Guangzhou, Guangdong 510120 China; 3 Departments of Surgery and Oncology, Johns Hopkins Medical Institutions, Blalock #607, 600 N. Wolfe St, Baltimore, Maryland 21287 USA

**Keywords:** Breast cancer, Nomogram, Lymph node status

## Abstract

**Background:**

Prediction of axillary lymph node (ALN) status preoperatively is critical in the management of breast cancer patients. This study aims to develop a new set of nomograms to accurately predict ALN status.

**Methods:**

We searched the National Cancer Database to identify eligible female breast cancer patients with profiles containing critical information. Patients diagnosed in 2010–2011 and 2012–2013 were designated the training (*n* = 99,618) and validation (*n* = 101,834) cohorts, respectively. We used binary logistic regression to investigate risk factors for ALN status and to develop a new set of nomograms to determine the probability of having any positive ALNs and N2–3 disease. We used ROC analysis and calibration plots to assess the discriminative ability and accuracy of the nomograms, respectively.

**Results:**

In the training cohort, we identified age, quadrant of the tumor, tumor size, histology, ER, PR, HER2, tumor grade and lymphovascular invasion as significant predictors of ALNs status. Nomogram-A was developed to predict the probability of having any positive ALNs (P_any) in the full population with a C-index of 0.788 and 0.786 in the training and validation cohorts, respectively. In patients with positive ALNs, Nomogram-B was developed to predict the conditional probability of having N2–3 disease (P_con) with a C-index of 0.680 and 0.677 in the training and validation cohorts, respectively. The absolute probability of having N2–3 disease can be estimated by P_any*P_con. Both of the nomograms were well-calibrated.

**Conclusions:**

We developed a set of nomograms to predict the ALN status in breast cancer patients.

**Electronic supplementary material:**

The online version of this article (doi:10.1186/s12885-017-3535-7) contains supplementary material, which is available to authorized users.

## Background

Treatment for early-stage breast cancer is focused on minimizing axillary surgery. The IBCSG 23–01 trial [1] demonstrated that patients with micrometastases in sentinel lymph nodes (SLNs) can be spared from axillary lymph node dissection (ALND). Furthermore, ALND does not provide any additional benefit in patients who received breast-conserving surgery (BCS) with 1–2 positive SLNs, as demonstrated in the Z11 trial [2]. Ongoing studies [3–5] are attempting to extend the results reported in the Z11 trial to mastectomy patients. The SOUND trial and the recent NCT01821768 trial [6] have been designed to explore the possibility of abandoning SLNB in a select group of patients [7]. However, the safety of the selection criteria used in these studies is unconfirmed. Predictive models for axillary lymph node (ALN) status would help to identify patients who are more likely to have negative ALNs to spare SLNB. These models, presented as nomograms, were reported and validated in different populations [8–11]. However, none has been widely accepted in clinical practice, possibly due to the lack of external validation in a large population.

In addition, most of the reported models were designed to predict the probability of having any positive ALNs (≥ 1 positive ALNs). It is also important to predict the probability of having N2–3 disease (>/=4 positive ALNs) for clinical decision making. For example, in patients who fit the Z11 criteria and did not receive ALND, successful prediction of the axillary tumor burden may be informative for radiation oncologists in the determination of radiation fields.

The National Cancer Database (NCDB) is a joint program of the American College of Surgeons and the American Cancer Society. The database includes more than 1500 cancer programs in the United States with detailed tumor pathology information and overall survival data. Since 2010, data concerning HER2 status and lymphovascular invasion (LVI) have been available in the NCDB. In this study, we used data from the NCDB to develop novel and accurate nomograms that can predict the probability of having any positive ALNs and N2–3 disease. The wide range of patients represented in the NCDB may help to improve the robustness and generalizability of the novel nomograms.

## Methods

### Patient selection

We searched the NCDB registry dataset between 2010 and 2013 and identified female breast cancer patients using the following criteria:

### Inclusion criteria

1. Year of diagnosis ≥2010 (LVI and HER2 status have been available since 2010)
2. Female gender
3. A known number of lymph nodes was examined, and a known number of positive ALNs was reported
4. The location of the tumor was known (PRIMARY_SITE coding: C501;C502;C503; C504;C505)

### Exclusion criteria

1. T-stage unknown, DCIS or T4 patients, or tumor size larger than 10 cm.
2. Phyllodes tumor
3. Presence of metastatic disease at the time of diagnosis
4. Neoadjuvant chemotherapy
5. Patients with a prior tumor diagnosis
6. Patients with radical mastectomy, extended radical mastectomy or unknown surgery type
7. Bilateral breast cancer
8. Patients with overlapping lesions of the breast, multicentric lesions, or lesions that involved the entire breast (PRIMARY_SITE coding: C508;C509)
9. Tumor grade unknown, except for lobular carcinoma
10. ER, PR, and HER2 status unknown; HER2 borderline patients were also excluded
11. Unknown LVI status.

This was a retrospective study using anonymous and de-identified data from the NCDB. The authors cannot assess the information that could identify individual participants; therefore, this study was exempt from the Johns Hopkins Medicine Institutional Review Board and the Sun Yat-sen Memorial Hospital ethical committee review, and no consent was required.

### Statistical analysis

Patients diagnosed from 2010 to 2011 and from 2012 to 2013 with ≥1 nodes examined were defined as the training cohort and validation cohort, respectively, for predictive model development and validation.

We used the Chi-square test to identify risk factors for positive ALNs. The statistically significant (*P* < 0.001) risk factors were considered to be potential predictors of ALNs status and were all included in the full model. We used a binary logistic regression model to develop a predictive model for ALN status. We used Akaike information criterion (AIC) and ROC analysis to identify the optimal model. We used the full population to develop a prediction model (Model-A) of the risk of having any ALNs(+). Next, we developed a model (Model-B) that could estimate the conditional probability of having pN2–3, given the conditions that the patients had ALNs(+), that patients were ALN-positive, and that patients with <10 ALNs examined and > = 1 positive ALNs (*N* = 23,106) were excluded.

We used the “rms” package of the R software to develop nomograms to visualize our predictive model graphically. Nomogram-A estimated the probability of having any positive ALNs (P_any). Nomogram-B estimated the conditional probability of having pN2–3 disease (P_con). The probability of having pN2–3 disease can be calculated as P_any*P_con.

We used the ROC analysis and calibration plots to evaluate the discriminative ability and accuracy of the models, respectively. The performance of the models were evaluated and validated internally in the training cohort and externally in the validation cohort, respectively.

For sensitivity analysis, we randomly selected 500, 5000 and 50,000 patients from the study population and calculated the AUC values of the model in these sub-populations. We repeated the sampling for *N* = 200 times and calculated the mean and standard deviations of the AUC values to determine the stability of AUC values.

All of the statistical analyses were performed using STATA 13.0MP and R.

## Results

### Clinicopathological features

This study included 201,452 breast cancer patients cataloged in the NCDB with a median age of 61 years old. The clinicopathological features are listed in Table 1. There were 99,618 and 101,834 patients in the training and the validation cohort, respectively. Patient features were similar between the training cohort and the standard validation cohort.

**Table 1:** Clincopathological features of the study populations

	Training Cohort		Validation Cohort	
	N	%	N	%
Year Of Diagnosis				
2010	47,203	47.38	0	0.00
2011	52,415	52.62	0	0.00
2012	0	0.00	50,965	50.05
2013	0	0.00	50,869	49.95
Age Group				
Age < =50Yrs	23,231	23.32	22,203	21.80
50-60Yrs	25,967	26.07	26,600	26.12
> 60Yrs	50,420	50.61	53,031	52.08
Location Of Lesions				
UIQ	18,891	18.96	19,939	19.58
UOQ	53,372	53.58	54,995	54.00
LOQ	11,425	11.47	11,776	11.56
LIQ	9086	9.12	8776	8.62
Central	6844	6.87	6348	6.23
Race				
White	84,246	84.57	85,967	84.42
African American	10,334	10.37	10,429	10.24
Others	4184	4.20	4568	4.49
Unknown	854	0.86	870	0.85
T-Stage				
T1^a^	69,375	69.64	71,740	70.45
T2	27,675	27.78	27,528	27.03
T3	2568	2.58	2566	2.52
N-Stage				
N0	73,662	73.94	76,954	75.57
N1	19,724	19.80	19,362	19.01
N2	4313	4.33	3829	3.76
N3	1919	1.93	1689	1.66
Histology				
IDC	75,974	76.27	77,806	76.40
ILC	8582	8.61	9795	9.62
IDC & ILC	5005	5.02	5076	4.98
IDC & Others	3359	3.37	3390	3.33
IMC	1849	1.86	1857	1.82
Others	4849	4.87	3910	3.84
Grade				
I	25,663	25.76	26,780	26.30
II	43,908	44.08	45,673	44.85
III	29,420	29.53	28,304	27.79
Others/NA	627	0.63	1077	1.06
Estrogen Receptor				
Negative	15,746	15.81	14,902	14.63
Positive	83,872	84.19	86,932	85.37
Progesterone Receptor				
Negative	25,080	25.18	23,426	23.00
Positive	74,538	74.82	78,408	77.00
Her2				
Negative	87,670	88.01	92,043	90.39
Positive	11,948	11.99	9791	9.61
Lymphovascular Invasion				
Not Present	80,657	80.97	83,226	81.73
Present	18,961	19.03	18,608	18.27
Charlson-Deyo Score				
0	83,641	83.96	84,466	82.94
1	13,297	13.35	14,319	14.06
2	2680	2.69	3049	2.99
Breast Surgery				
BCS + RT	64,552	64.80	66,480	65.28
Mastectomy^b^	35,066	35.20	35,354	34.72

*NCDB* national cancer database, *Yrs* years, *HER2* human epidermal growth factor receptor 2, *BCS* breast-conserving surgery, *RT* radiotherapy, *LIQ* lower-inner quadrant, *LOQ* lower-outer quadrant, *UIQ* Upper-inner quadrant, *UOQ* Upper-outer quadrant, *NA* not available, *IDC* infiltrating ductal carcinoma, *ILC* infiltrating lobular carcinoma, *IMC* invasive mucinous carcinoma;

^a^DCIS with micrometastasis (T1mic) were included in T1

^b^Subcutaneous mastectomy and reconstruction surgery were included

### Nomogram for predicting risk of any positive ALNs

**Fig. 1:**
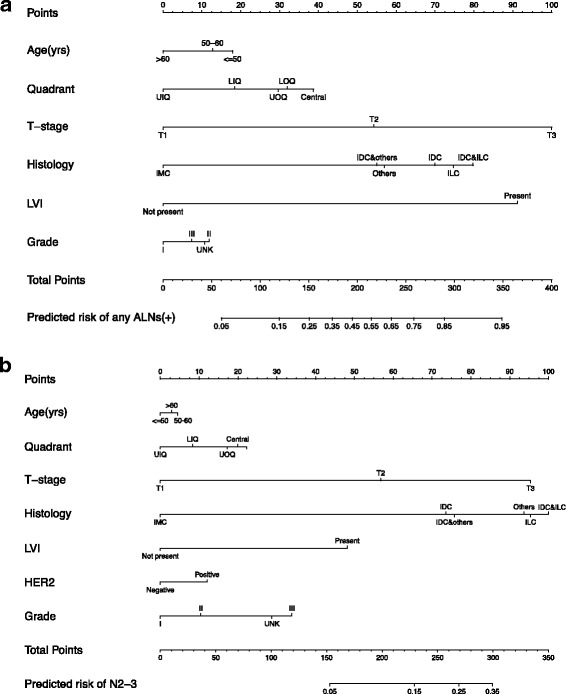
**a** Nomogram to predict the probability of having any positive ALNs (P_any); **b** Nomogram to predict the conditional probability of having N2–3 disease (P_con), when the patients have any positive ALNs. The absolute probability of having N2–3 can be estimated by P_any*P_con

We used Chi-square analysis and logistic regression as univariate and multivariate analysis to evaluate the risk factors for any positive ALNs in the training cohort. Age, location of lesions, T-stage, histology, ER, PR, HER2, tumor grade and LVI were independent predictors for any positive ALNs by univariate analysis (Table 2). These variables were further confirmed as independent factors in the multivariate analysis, and variables were incorporated in the full model. We also tested some variant models with different variables included. The full model had similar AIC and C-index with the variant model 2 (Additional file 1 : Table S1) and the latter consisted of fewer variables. Therefore, we selected variant model 2 (with age, quadrant, size, histology, grade and LVI as predictors) for development of nomogram A to predict the risk of any positive ALNs (Fig. 1).

**Table 2:** Analysis of risk factors for any positive ALNs

	Univariate analysis					Multivariate analysis	
	N0		N1–3		*P* ^b^	OR(95%)	*P*
	n	%^a^	n	%^a^			
Age Group							
Age < =50Yrs	15,652	67.38	7579	32.62	<0.001	1	
50-60Yrs	18,757	72.23	7210	27.77		0.91(0.87–0.95)	<0.001
> 60Yrs	39,253	77.85	11,167	22.15		0.70(0.67–0.73)	<0.001
Location Of Lesions							
UIQ	15,519	82.15	3372	17.85	<0.001	1	
UOQ	38,406	71.96	14,966	28.04		1.84(1.75–1.93)	<0.001
LOQ	8149	71.33	3276	28.67		1.91(1.79–2.03)	<0.001
LIQ	7089	78.02	1997	21.98		1.46(1.36–1.56)	<0.001
Central	4499	65.74	2345	34.26		2.19(2.03–2.35)	<0.001
T-Stage							
T1	57,744	83.23	11,631	16.77	<0.001	1	
T2	15,160	54.78	12,515	45.22		3.05(2.95–3.16)	<0.001
T3	758	29.52	1810	70.48		7.85(7.12–8.65)	<0.001
Histology							
IDC	55,824	73.48	20,150	26.52	<0.001	1	
ILC	6174	71.94	2408	28.06		1.08(1.02–1.15)	0.009
IDC & ILC	3437	68.67	1568	31.33		1.19(1.11–1.28)	<0.001
IDC & Others	2688	80.02	671	19.98		0.74(0.67–0.82)	<0.001
IMC	1731	93.62	118	6.38		0.24(0.19–0.29)	<0.001
Others	3808	78.53	1041	21.47		0.79(0.73–0.86)	<0.001
Grade							
I	21,746	84.74	3917	15.26	<0.001	1	
II	32,129	73.17	11,779	26.83		1.29(1.23–1.35)	<0.001
III	19,337	65.73	10,083	34.27		1.31(1.24–1.38)	<0.001
Others/NA	450	71.77	177	28.23		1.27(1.04–1.56)	0.022
Estrogen Receptor							
Negative	11,326	71.93	4420	28.07	<0.001	1	
Positive	62,336	74.32	21,536	25.68		1.20(1.12–1.28)	<0.001
Progesterone Receptor							
Negative	18,233	72.70	6847	27.30	<0.001	1	
Positive	55,429	74.36	19,109	25.64		1.16(1.10–1.23)	<0.001
Her2							
Negative	65,656	74.89	22,014	25.11	<0.001	1	
Positive	8006	67.01	3942	32.99		1.11(1.05–1.16)	<0.001
Lymphovascular Invasion							
Not Present	66,799	82.82	13,858	17.18	<0.001	1	
Present	6863	36.20	12,098	63.80		6.36(6.12–6.60)	<0.001

*ALN* axillary lymph node, *Yrs* years old, *HER2* human epidermal growth factor receptor 2, *LIQ* lower-inner quadrant, *LOQ* lower-outer quadrant, *UIQ* Upper-inner quadrant, *UOQ* Upper-outer quadrant, *N/A* not available, *IDC* infiltrating ductal carcinoma, *ILC* infiltrating lobular carcinoma, *IMC* invasive mucinous carcinoma, *NS* non-significant

^a^Row percentage was shown

^b^Chi-square test was used for univariate analysis

### Nomogram for predicting pN2–3 disease in patients with any positive ALNs

We excluded patients with negative ALNs to predict the pN2–3 disease in patients with any positive ALNs. Patients had <10 ALNs nodes examined, and ≥1 positive ALNs were also excluded (*N* = 23,106). Univariate analysis suggested that age, location of lesions, T-stage, histology, ER, PR, HER2, tumor grade and LVI were risk factors for pN2–3 disease in patients with any positive ALNs (Table 3). These variables, except for ER and PR status, were confirmed as independent risk factors in the multivariate analysis. The full model was selected based on its lowest AIC and the highest C-index (Additional file 1 : Table S1). Nomogram-B (Fig. 1) was developed to predict the conditional probability of having pN2–3 patients, given that patients have ≥1 positive ALNs.

**Table 3:** Analysis of risk factors for pN2–3^a^

	Univariate analysis					Multivariate analysis	
	N0		N1–3		*P* ^b^	OR(95%)	*P*
	n	%^a^	n	%+			
Age Group							
Age < =50Yrs	3229	66.30	1641	33.70	<0.001	1	
50-60Yrs	2958	66.20	1510	33.80		1.06(0.97–1.16)	0.202
> 60Yrs	3917	62.49	2351	37.51		1.23(1.14–1.34)	<0.001
Location Of Lesions							
UIQ	1374	69.39	606	30.61	<0.001	1	
UOQ	5753	64.16	3213	35.84		1.30(1.17–1.45)	<0.001
LOQ	1300	63.82	737	36.18		1.38(1.20–1.59)	<0.001
LIQ	788	69.31	349	30.69		1.15(0.98–1.36)	0.095
Central	889	59.83	597	40.17		1.36(1.17–1.58)	<0.001
T-Stage							
T1	4589	76.36	1421	23.64	<0.001	1	
T2	4993	60.53	3256	39.47		1.83(1.69–1.97)	<0.001
T3	522	38.75	825	61.25		3.97(3.48–4.52)	<0.001
Histology							
IDC	8027	66.32	4076	33.68	<0.001	1	
ILC	761	52.02	702	47.98		1.96(1.73–2.22)	<0.001
IDC & ILC	570	61.16	362	38.84		1.27(1.10–1.47)	0.001
IDC & Others	272	67.83	129	32.17		0.91(0.73–1.13)	0.396
IMC	59	81.94	13	18.06		0.44(0.24–0.82)	0.01
Others	415	65.35	220	34.65		1.02(0.85–1.21)	0.848
Grade							
I	1482	75.88	471	24.12	<0.001	1	
II	4433	65.51	2334	34.49		1.30(1.15–1.47)	<0.001
III	4140	60.98	2649	39.02		1.44(1.26–1.64)	<0.001
Others/NA	49	50.52	48	49.48		1.48(0.95–2.30)	0.08
Estrogen Receptor							
Negative	1892	62.18	1151	37.82	<0.001	1	
Positive	8212	65.37	4351	34.63		1.05(0.92–1.19)	0.467
Progesterone Receptor							
Negative	2809	61.93	1727	38.07	<0.001	1	
Positive	7295	65.90	3775	34.10		0.93(0.84–1.04)	0.219
Her2							
Negative	8541	65.96	4408	34.04	<0.001	1	
Positive	1563	58.83	1094	41.17		1.30(1.18–1.42)	<0.001
Lymphovascular Invasion							
Not Present	5632	74.12	1966	25.88	<0.001	1	
Present	4472	55.84	3536	44.16		2.11(1.96–2.26)	<0.001

*ALN* axillary lymph node, *Yrs* years old, *HER2* human epidermal growth factor receptor 2, *LIQ* lower-inner quadrant, *LOQ* lower-outer quadrant, *UIQ* Upper-inner quadrant, *UOQ* Upper-outer quadrant, *N/A* not available, *IDC* infiltrating ductal carcinoma, *ILC* infiltrating lobular carcinoma, *IMC* invasive mucinous carcinoma, *NS* non-significant

^a^Only patients with positive nodes were included. Patients with <10 axillary lymph nodes examined <10 but >1 positive ALNs were excluded

^b^Row percentage was shown.

***Chi-square test was used for univariate analysis

### Distribution of the predicted probability

The training cohort and the validation cohort exhibited a similar distribution of predicted risks by the new model (Additional file 2 : Fig. S1). Most of the predicted risk of any ALNs ranged between 0 and 20%. Most of the predicted risks of pN2–3 disease ranged between 10% and 50% and between 0% and 10% in patients with any positive ALNs and in all populations, respectively.

### Validation of the nomograms

**Fig. 2:**
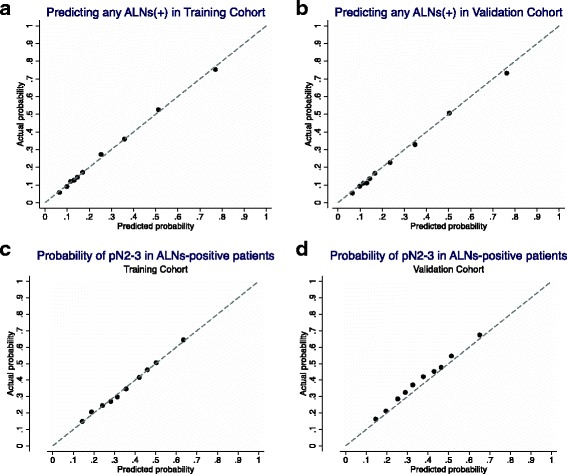
Calibration plots of nomogram-A to predict the probability of having any positive ALNs in the **a**) training and **b**) validation cohort, and nomogram-B to predict the conditional probability of having N2–3 disease in the **c**) training and **d**) validation cohort

The AUC values of the nomograms (Additional file 1 : Table S1) for predicting any positive ALNs and pN2–3 disease were 0.788 and 0.680 in the training cohort and 0.786 and 0.677 in the validation cohort, respectively. The calibration plot (Fig. 2) suggested that the nomograms were well-calibrated. The average estimation errors of predicting any positive ALNs and pN2–3 disease were 0.78% and 0.85% in the training cohort and 1.14% and 2.79% in the validation cohort, respectively.

### Sensitivity analysis

For sensitivity analysis, we randomly selected 500, 5000 and 50,000 patients from the training and validation cohorts and performed the ROC analysis and calibration plot analysis. We repeated the re-sampling 200 times to obtain a reliable estimation of the AUC values and average prediction error between the actual and predicted risks. As shown in Additional file 3 : Table S2, the estimated AUC values and average prediction error were similar among sub-populations with varied sample sizes.

## Discussion

### Accuracy of the nomograms

The first predictive model for ALN status was developed a decade ago by Bevilacqua et al. [8]. The authors retrospectively reviewed the database of MSKCC and identified 3786 and 1545 breast cancer patients as training and validation sets, respectively. A nomogram was developed using age, tumor size, special pathology type, location, LVI, multifocal status, nuclear grade, and ER and PR status as predictors of ALN status. Chen et al. [10] validated the MSKCC model in a Chinese population (*n* = 1545) and reported a new nomogram (the Shanghai model) using data from Chinese breast cancer patients. However, the MSKCC model did not incorporate HER2 status. Reyal et al. [11] reported that molecular subtype approximation, including ER, PR and HER2, is also a determinant of ALN status, and another nomogram was later developed (the Paris model). Additionally, several more models [9, 12–15] have been developed to predict ALN status. However, none of these models has been widely accepted by treatment guidelines, and clinical practice has not significantly changed. A lack of sufficient evidence to support external validity is one of the major underlying reasons. In addition, these models can only predict the risk of having ≥1 positive ALN. In the current study, we used a large multi-institutional NCDB population to develop and validate a set of nomograms that can predict the risk of having any positive ALNs and N2–3 disease.

### Benefit of the new nomograms in the post-Z0011 era

The Z11 study [2] demonstrated that patients with 1–2 positive SLNs receiving BCS and standardized adjuvant therapies could be spared from ALND [16]. However, it is impossible to know whether a patient fits the Z11 criteria or not before surgery, as the number of positive SLNs can only be identified during or after surgery. Our nomograms may be able to identify patients who may not fit the Z11 criteria by predicting the risk of having N2–3 disease preoperatively. If a patient had a high risk of having N2–3 disease, she may be unlikely to fit the Z11 criteria.

Because mastectomy patients were not included in the Z11 study, ALND is still a routine procedure for SLN-positive patients. However, several retrospective studies suggested the feasibility to omit ALND in selected mastectomy patients with positive [4, 17, 18]. A prospective randomized trial was also initiated to test this hypothesis (NCT02112682). Therefore, the trend that Z11 conclusions could be extended to mastectomy patients is very clear, and with the help of these nomograms, surgeons may feel safer in omitting the ALND in selected mastectomy patients with positive SLNs.

One concern related to omitting the ALND in mastectomy patients is whether RT should be given. The NCCN guidelines [19] clearly recommend RT to the infraclavicular region, supraclavicular area, and internal mammary nodes for patients with N2–3 disease (≥4 positive ALNs). For patients with 1–3 positive nodes (N1 disease) after mastectomy, radiotherapy coverage of these areas was considered controversial by NCCN panel members [19] because high-level contradictory evidence was apparent [20–23]. With our nomograms, omitting ALND in selected mastectomy patients after positive SLNs may not be a major problem, as the radiation oncologist can estimate the risk of having N2–3 disease and determine the treatment plans. Additionally, these nomograms would be more helpful to the radiation oncologists, in that 1) they may help reassure them that patients who met Z11 criteria do not need additional radiation therapy, in terms of increasing the tangents/fields for radiation; 2) For patients who received neoadjuvant chemotherapy, RT decisions should be based on pre-chemotherapy tumor features regardless of the tumor response [18]. Our nomograms may be useful in the estimation of axillary tumor burden prior to the initiation of neoadjuvant chemotherapy to provide more information.

### Benefit of the new nomograms in the “SLNB-sparing” era

In the post-Z11 era when “the days are numbered for axillary surgery” [24], it is likely that SLNB could be omitted in selected patients. The SOUND trial [7], proposed in 2012, was designed to test this hypothesis. In the SOUND trial, T1 breast cancer patients with clinically negative axilla were randomized into groups receiving either observation or SLNB. There were only 12.8% of patients with positive ALNs in the SLNB group, suggesting the high probability that SLNB could be spared in the future. The development of the nomograms is consistent with the trend towards the “SLNB-sparing” era. If we could identify node negative patients preoperatively, the omission of SLNB would be much safer, without the need to wait for the results of the SOUND trial. Sparing SLNB would improve the quality of life and reduce the medical cost and all possible surgical complications. The authors recently reported that the physical function of the upper limb in the no-SLNB group was significantly better than in the SLNB-group, suggesting the benefit of minimizing axillary surgery when appropriate [25].

In the “SLNB-sparing” era, if the SOUND trial demonstrated that not performing SLNB in selected patients is safe, there will be concerns regarding the absence of axillary staging on the decision to use adjuvant therapies. For example, post-mastectomy radiotherapy (PMRT) is not necessary in T1–2 patients with negative ALNs, whereas in patients with positive ALNs, PMRT is strongly recommended by the NCCN guidelines [19]. The need for radiotherapy has also influenced the optimal timing of breast reconstruction (e.g., immediate vs. delayed). Additionally, a T1a patient with HER2 positive disease may be spared from chemotherapy if ALNs were negative, and adjuvant chemotherapy is recommended for patients with positive ALNs [19]. Taken together, the ability to predict the probability of having any positive ALNs (or N0 disease) would be helpful in the “SLNB-sparing” era in the future.

### Limitations

There are several limitations to this study.

First, several of the predictors of these models, such as LVI and multifocal lesions, may not always be available prior to surgery. Core needle biopsy may not provide an adequate volume of tissue for the identification of LVI. These issues may limit the utility of the models developed in the current study. However, ultrasound-guided vacuum-assisted biopsy [26, 27] has been used by many institutions and may provide a larger volume of tissue for the identification of LVI. Current imaging modalities can provide an accurate estimate of tumor size and multifocality [28–31].

Second, several important variables are not available in the NCDB, such as whether the tumor was palpable or ki-67 status. More importantly, the clinical axillary status was also not available in this study. In patients with clinically negative axilla, the probability of having N2–3 disease is very low. The performance of the model in these patients needs to be validated.

Third, the NSABP B-32 trial suggested a 10% false-negative rate of SLNs. In our study, patients with SLNB only, without any positive SLNs, were classified as having no positive ALNs. This limitation cannot be avoided when using data from the modern era when SLNB is the routine practice. However, we believe that the 10% false-negative rate may not significantly affect the performance of our model.

Fourth, when developing nomogram-B for predicting the conditional probability of having N2–3 disease, we excluded 23,106 patients (11.5%, 23,106/201,452) with ≥1 positive ALNs but with less than 10 ALNs evaluated. We may have skewed the data by excluding these patients. However, we considered that the benefit of excluding these patients might outweigh the harm of including them. As demonstrated in the Z11 study, patients with 1–2 SLN+ without further ALND may theoretically have a 27% risk of additional positive ALNs. Therefore, the exact amount of positive ALNs in these patients was unknown, leading to the inaccuracy of model development and validation.

Fifth, these nomograms can only be used in patients with a single focus of disease and only in patients with unilateral disease.

## Conclusions

In this study, we used a large multi-institutional NCDB population to develop a set of nomograms to predict nodal status in breast cancer patients. Future validation studies are needed to confirm our findings.

## Additional files

Additional file 1: Table S1. Model performance of the full models and the variants. This table showed the performance of the full models and their variants. (XLSX 42 kb)

Additional file 2: Figure S1. Kernel density plots of the a) predicted probability of having any positive ALNs by nomogram-A, b) predicted conditional probability of having N2–3 disease in patients with positive ALNs by nomogram-B, and c) the predicted absolute probability of having N2–3 disease in all populations, by nomogram-A and B. (PDF 54 kb)

Additional file 3: Table S2. Sensitivity analysis of the model performance in population with different sample sizes. We used different populations with different sample size to assess the performance and the robustness of the models. (XLSX 41 kb)

## Abbreviations

AIC: Akaike information criterion
ALN: Axillary lymph node
ALND: Axillary lymph node dissection
AUC: Area under the curve
BCS: Breast conserving surgery
ER: Estrogen receptor
HER2: Human epidermal growth receptor 2
LVI: lymphovascular invasion
NCDB: National Cancer Database
PMRT: Post-mastectomy radiotherapy
PR: Progesterone receptor
ROC: Receiver-operator-curve
SLN: Sentinel lymph node
