# Supplementary material for: Development of nomograms to predict axillary lymph node status in breast cancer patients
Source: BMC Cancer. 2017 Aug 23;17:561. doi: 10.1186/s12885-017-3535-7 (PMC5569510; doi:10.1186/s12885-017-3535-7)
Supplement: Supplementary file 2 — Kernel density plots of the a) predicted probability of having any positive ALNs by nomogram-A, b) predicted conditional probability of having N2–3 disease in patients with positive ALNs by nomogram-B, and c) the predicted absolute probability of having N2–3 disease in all populations, by nomogram-A and B. (PDF 54 kb) [file 12885_2017_3535_MOESM2_ESM.pdf]

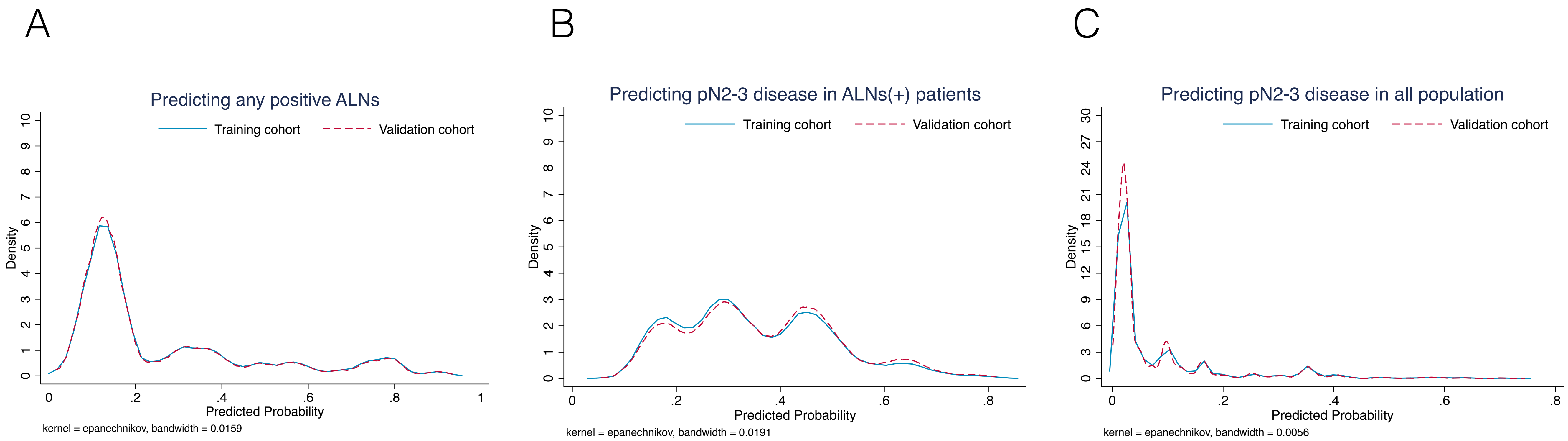
A
Predicting any positive ALNs
Training cohort
Validation cohort
Density
0 1 2 3 4 5 6 7 8 9 10
0 .2 .4 .6 .8 1
Predicted Probability
kernel = epanechnikov, bandwidth = 0.0159
B
Predicting pN2-3 disease in ALNs(+) patients
Training cohort
Validation cohort
Density
0 1 2 3 4 5 6 7 8 9 10
0 .2 .4 .6 .8
Predicted Probability
kernel = epanechnikov, bandwidth = 0.0191
C
Predicting pN2-3 disease in all population
Training cohort
Validation cohort
Density
0 3 6 9 12 15 18 21 24 27 30
0 .2 .4 .6 .8
Predicted Probability
kernel = epanechnikov, bandwidth = 0.0056
